# Effects of in vivo repositioning of slim modiolar electrodes on electrical thresholds and speech perception

**DOI:** 10.1038/s41598-021-94668-6

**Published:** 2021-07-23

**Authors:** Sang-Yeon Lee, Young Seok Kim, Hyung Dong Jo, Yoonjoong Kim, Marge Carandang, Gene Huh, Byung Yoon Choi

**Affiliations:** 1 Department of Otorhinolaryngology-Head and Neck Surgery, Seoul National University Hospital, Seoul, South Korea; 2 Department of Otorhinolaryngology-Head and Neck Surgery, Seoul National University Bundang Hospital, Seongnam, South Korea; 3 Department of Otorhinolaryngology-Head and Neck Surgery, Chungnam National University Hospital, Daejeon, South Korea; 4 Department of Otorhinolaryngology-Head and Neck Surgery, East Avenue Medical Center, Metro Manila, Philippines

**Keywords:** Diseases, Health care, Medical research, Signs and symptoms

## Abstract

The slim modiolar electrode has been reported to ensure better modiolar proximity than previous conventional perimodiolar electrodes and consistently high scala tympani localization. Nonetheless, variability in modiolar proximity exists even among slim modiolar electrodes, still leaving room for further improvement of modiolar proximity, which may positively affect functional outcomes. Given this, the pull-back maneuver was reported to increase the modiolar proximity of slim modiolar electrodes in a cadaveric study, but in vivo repositioning effects remain to be established. Here we identified that the pull-back maneuver led to better modiolar proximity than conventional insertion while maintaining a similar angular insertion depth. Notably, the reduced electrode-modiolus distance from the pull-back maneuver was associated with significantly lower impedances across electrodes postoperatively as well as reduced intraoperative electrophysiological thresholds than conventional insertion. Among adult cochlear implant recipients, this maneuver resulted in significantly better sentence recognition scores at three months postoperatively when compared to those with a conventional insertion; however, this benefit was not observed at later intervals. Collectively, slim modiolar electrodes with the pull-back maneuver further enhance the modiolar proximity, possibly leading to better open-set sentence recognition, at least in the early postoperative stage.

## Introduction

The slim modiolar electrode (e.g., CI532 or CI632) (Cochlear; Sydney, Australia) has been reported to ensure better modiolar proximity than other currently available electrodes while reducing intracochlear trauma^1–10^. Modiolar distance and scalar translocation of the electrodes have been suggested as significant positional determinants of hearing outcomes for conventional perimodiolar electrodes, while the depth of insertion was the most significant factor for straight electrode arrays^11,12^. Better modiolar proximity has been shown to yield more specific electrical stimulation with a smaller spread of excitation^1,13^, leading to better electrode discrimination capability and potentially better speech perception performance^14,15^.

Indeed, there has been recent growing evidence that this new slim modiolar electrode significantly enhanced the efficiency of neural-electrode interaction by reducing electrode modiolar distance (EMD), as evidenced by decreased telemetry threshold levels^16^, as well as improved battery efficiency^17,18^, outdoing conventional perimodiolar electrodes. In addition, the slim structure with controlled insertion via a sheath deployment system enabled consistently higher scala tympani position than did the conventional perimodiolar electrodes^4,19,20^, leaving the degree of modiolar proximity an important adjustable positional determinant influencing speech outcome in slim modiolar electrodes. However, it is still true that modiolar proximity shows considerable variability even among patients implanted with slim modiolar electrodes^21^.

A growing body of evidence suggests that the enhanced modiolar proximity of slim modiolar electrodes compared with conventional perimodiolar electrodes would lead to improved place-pitch spectral discrimination^14^, and speech perception outcomes^19,22^. However, not all reports in the literature fully support this^8,23,24^, possibly due to presence of other influential/confounding factors, limited sample numbers for statistical significance, or heterogeneous degrees of modiolar proximity even among subjects with slim modiolar electrodes. Alternatively, outcome improvement due to reduction of EMD may have a saturation exactly at the level of EMD provided by conventional perimodiolar electrode, reaching the ceiling effect. Nevertheless, identifying maneuvers or adjustable parameters that could further improve the modiolar proximity of slim modiolar electrodes may help to better test the hypothesis that speech outcome inversely correlates with EMD.

Given this, a delicate surgical technique called the “pull-back maneuver” previously demonstrated in human cadaveric temporal bones to enhance the modiolar proximity of slim modiolar electrodes, merits special attention^25^. While advancing past the first marker (i.e., over-insertion) of the electrode array led to an increase in EMD, subsequently pulling back to the first white marker of the electrode could elicit significantly enhanced modiolar proximity without any changes in the total insertion depth^4^. These findings suggest that adjusting the marker position in and out across the round window using this surgical maneuver may affect the electrode position mediolaterally without advancing the tip anteriorly. Despite putative evidence of a link between the pull-back maneuver and better modiolar proximity for modiolar hugging electrodes^26–30^, in vivo effects of the pull-back maneuver on EMD and electrophysiological parameters related to slim modiolar electrodes are still lacking. Additionally, the effects on speech outcome due to the change in mediolateral position of electrodes after using this surgical maneuver has never been addressed, even for conventional perimodiolar electrodes, let alone slim modiolar electrodes. Here we explore the in vivo effect of the pull-back maneuver-assisted insertion of slim modiolar electrodes on modiolar proximity, electrophysiological thresholds, and speech perception. Through this study, we aim to reach optimal modiolar proximity by coupling the slim modiolar electrode with a complementary surgical maneuver.

## Results

### Demographics and clinical characteristics

**Figure 1:**
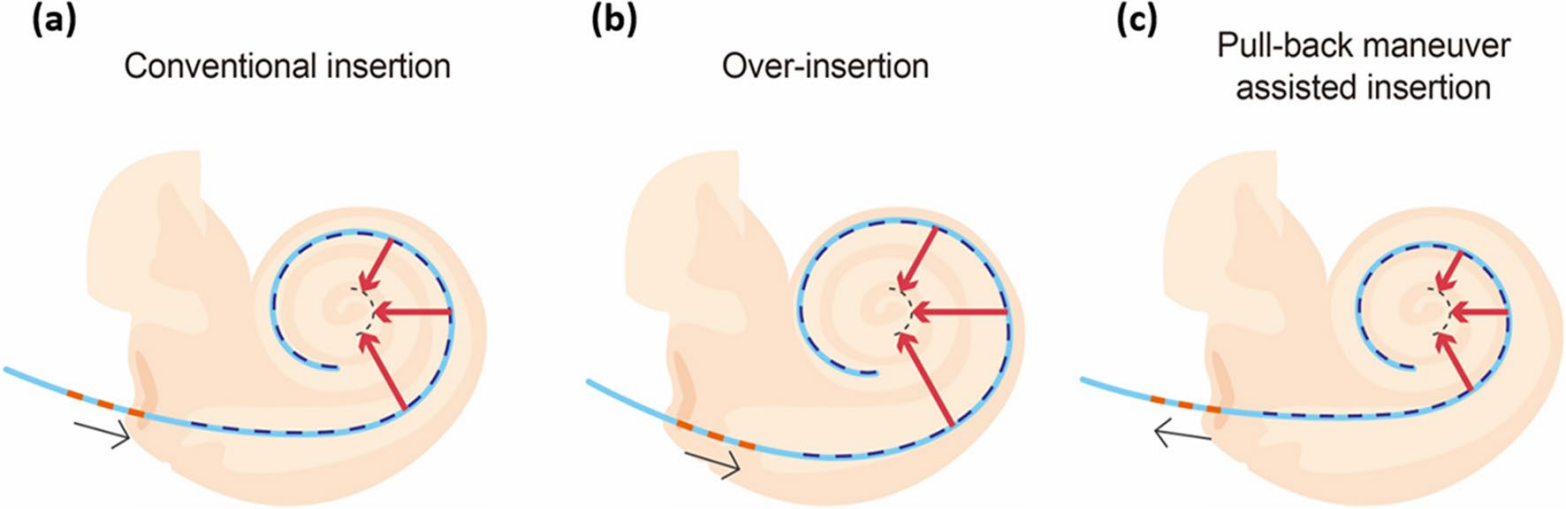
Schematic diagram of the degree of modiolar proximity of the slim modiolar electrode array depending on insertion technique. The slim modiolar electrode array can be advanced or retreated (black arrows) with reference to three white markers of the electrode array and their relationship with the round window. (**a**) Conventional insertion (first white marker), (**b**) over-insertion (up to third white marker), and (**c**) pull-back maneuver assisted insertion (first white marker). Red arrows indicate the distance between modiolus (dotted black line) and electrode array, reflecting the degree of modiolar proximity.

We conducted a retrospective study consisting of subjects who underwent implantation with a novel slim modiolar electrode (e.g., CI532 and CI632) performed by a single surgeon (B.Y.C.) through a round or extended round window approach. Ultimately, a total of 95 ears from 81 subjects with CI532 (N = 63, 76 ears) or CI632 (N = 18, 19 ears) implants were enrolled. Stratified by insertion techniques (Fig. 1), our cohort was subdivided into two groups: the conventional insertion (N = 36, 42 ears) and pull-back maneuver-assisted insertion groups (N = 45, 53 ears). The insertion technique was decided entirely in chronological order. Specifically, the first 42 and the next 53 ears were implanted using the ‘conventional’ and ‘pull-back’ maneuver, respectively. The clinical characteristics and demographics of each group are shown in Table 1. No significant difference in sex, laterality, or approach were observed between conventional insertion and pull-back maneuver groups. Following our criteria, no subject exhibited any definite cochlear anomalies, including cochlear nerve agenesis, based on radiological evaluations. A significant difference in the median age at cochlear implantation between the two groups was noted (Table 1). Additionally, we observed a positive correlation between age at cochlear implantation and cochlear duct length in this cohort (ρ = 0.365, *P* < 0.001 by Spearman’s rank-order correlation). However, cochlear duct length reflecting cochlear size did not differ between the conventional insertion and pull-back maneuver groups. Molecular genetic testing was conducted before cochlear implantation on all subjects, while congenital cytomegalovirus (cCMV) screening was conducted before cochlear implantation for subjects with congenital or prelingual deafness. The causative etiology for deafness was heterogeneous, regardless of groups.

**Table 1:** Demographics and clinical characteristics.

	Conventional insertion (N = 36, 42 ears)	Pull-back maneuver (N = 45, 53 ears)	*P* value
**Age at CI (years)**			
Median	5.5	43	0.005^a^
Range	1–90 (years)	1–91 (years)	
**Sex**			
Male	18	29	0.662^b^
Female	18	24	
**Laterality**			
Right	14	27	0.085^b^
Left	28	26	
**Cochlear size**			
Cochlear duct length	33.70 ± 0.26	34.14 ± 0.24	0.289^a^
**Approach**			
RW	39	53	0.083^b^
ERW	3	0	
**Etiology**			
Genetic variants	14	17	0.460^b^
cCMV infection	4	2	
Cochlear otosclerosis	1	1	
Cochlear nerve hypoplasia	2	0	
SSNHL	3	3	
Chronic otitis media	2	4	
Mobius syndrome with anotia	1	0	
Unknown	9	18	

The data are presented mean ± SEM.

Abbreviation: *N* number, *CI* cochlear implantation, *SEM* standard error of mean, *RW* round window, *ERW* extended round window, *cCMV* congenital cytomegalovirus, *SSNHL* sudden sensorineural hearing loss.

^a^That comparison of age at CI and cochlear duct length between two groups was based on the Mann Whitney U test due to non-standardized normality.

^b^That comparison of sex, laterality, electrode type, approach, and etiology between two groups was based on the Chi-square test and Fischer’s Exact test, as appropriate.

### Spiral diameter of electrode turns and angular insertion depth

**Figure 2:**
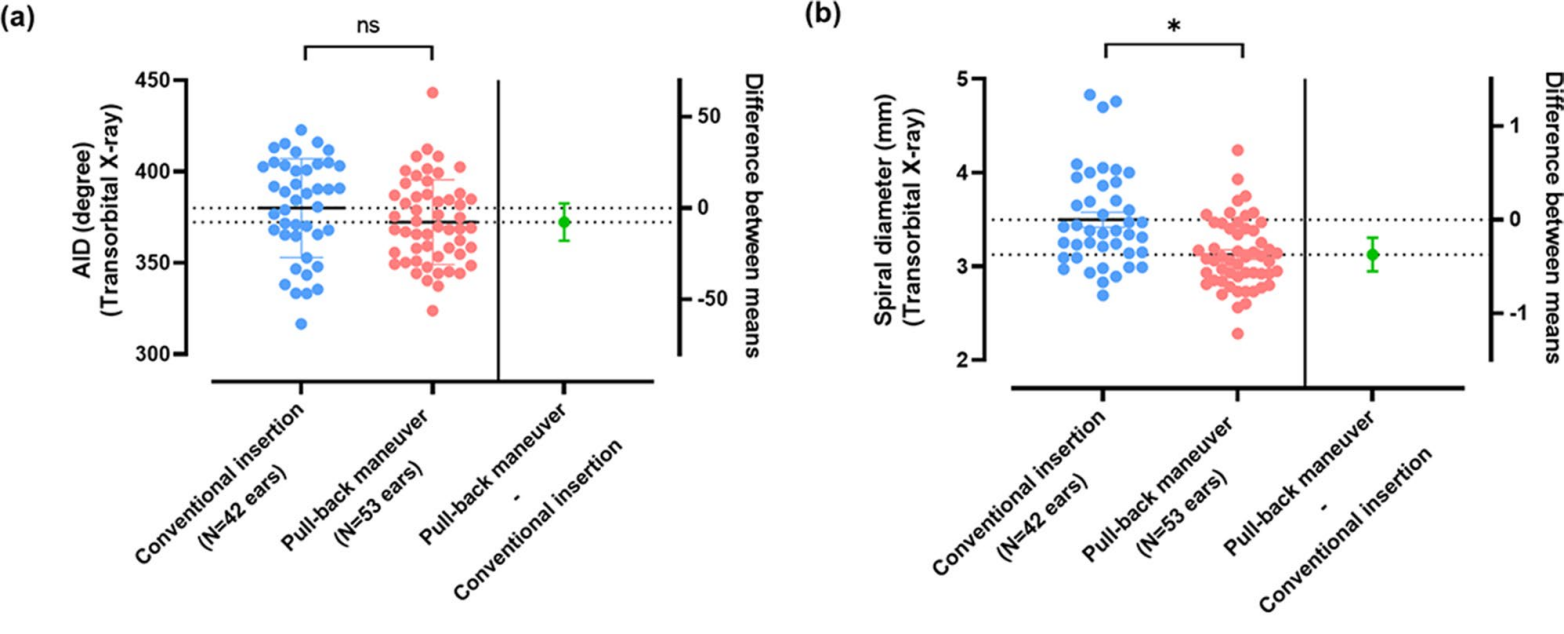
Comparison of spiral diameter and angular insertion depth between conventional insertion and pull-back maneuver groups. (**a**) The angular insertion depth is similar regardless of the insertion technique employed. The pull-back maneuver-assisted insertion did not elicit a significant decrease in angular insertion depth relative to that with conventional insertion. (**b**) The spiral diameter reflecting modiolar proximity was significantly different between insertion techniques. The pull-back maneuver group had significantly reduced spiral diameter as measured by transorbital X-ray, as compared with conventional insertion group. The pull-back maneuver-assisted insertion gives rise to enhancing modiolar proximity of slim modiolar electrodes in vivo. Data are means ± standard error of mean (SEMs). *P < 0.05 by independent t-test. *ns* no statistical significance.

The spiral diameter and angular insertion depth (AID) of the slim modiolar electrodes in situ on transorbital X-ray were compared between different insertion techniques. This pull-back maneuver did not elicit a significant difference in AID relative to conventional insertion (difference between means ± standard error of mean (SEM), 7.652 ± 5.165, *P* = 0.142 by independent t-test) (Fig. 2a). Furthermore, upon the intraoperative X-ray, tip fold-over of the electrode array was not noted in our cohort.

On the other hand, the insertion technique produced a significant difference in spiral diameter as measured from transorbital X-ray. The spiral diameter of the electrode turn was defined as the distance of the electrode turn measured on a horizontal line across the modiolus. The value of this X-ray-based measurement of the spiral diameter for evaluating modiolar proximity has been demonstrated by a tight correlation with a reliable and finely tuned indicator, the modified intracochlear positional index (ICPI), which evaluates the degree of modiolar proximity based on high-resolution computed tomography^31^. Specifically, the pull-back group (N = 53, 3.13 ± 0.37 mm) had a significantly shorter spiral diameter than the conventional insertion group (N = 42, 3.50 ± 0.51 mm, *P* < 0.001 by independent t-test) (Fig. 2b), showing the enhancing effect of the pull-back maneuver on modiolar proximity of slim modiolar electrodes in vivo*,* for the first time in literature. Overall, for slim modiolar electrodes, putting the electrode in and out in a straight line around the round window significantly influenced the final mediolateral position of the electrode relative to the modiolus.

### Electrically evoked compound action potential

**Figure 3:**
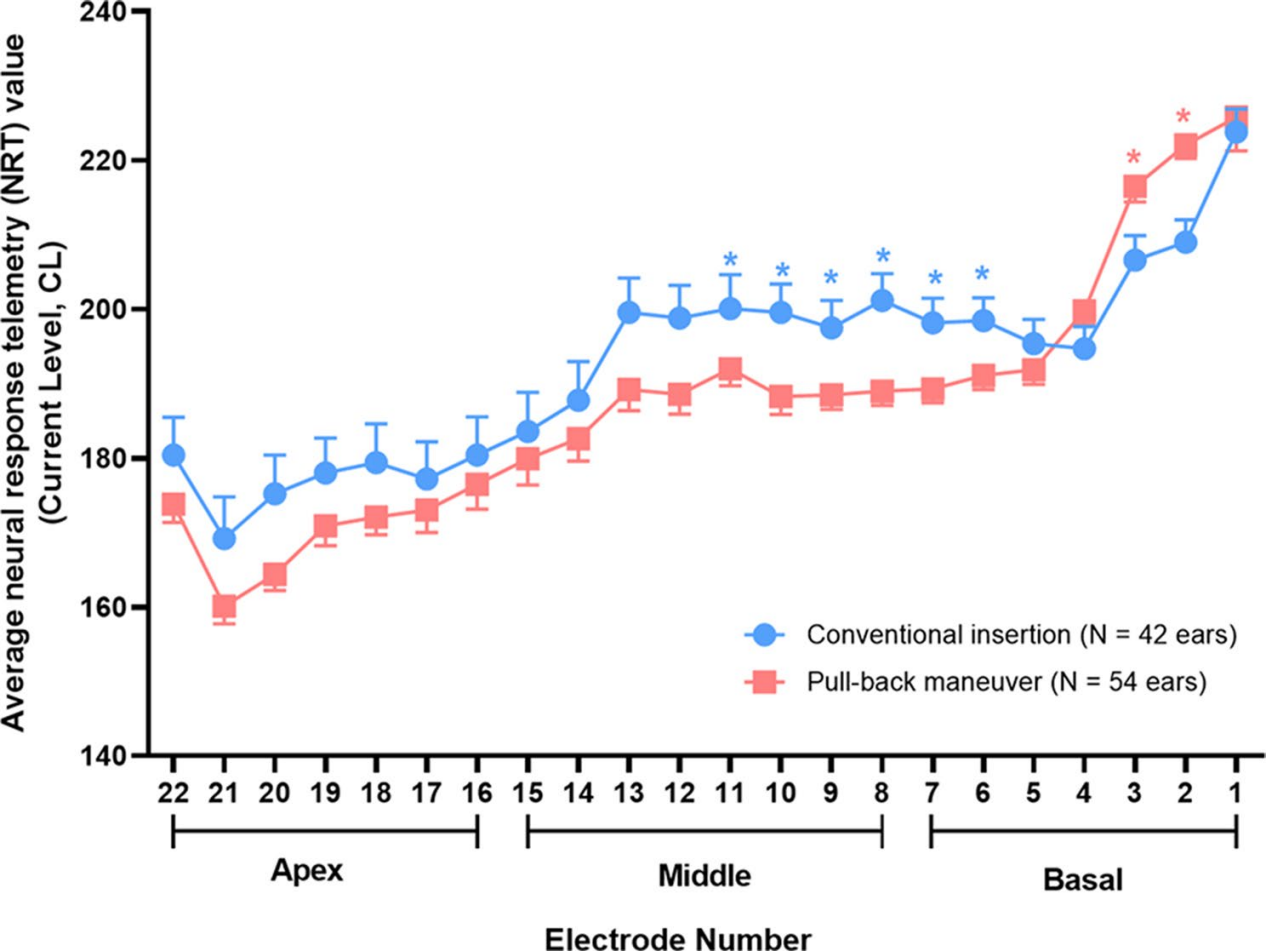
Intraoperative electrically evoked compound action potential (ECAP) thresholds according to insertion techniques. Using the pull-back maneuver-assisted insertion, the average ECAP thresholds of electrode channel is significantly lower, especially in the upper basal and middle cochlear region (Electrode 6–Electrode 12), as compared those using the conventional insertion. In contrast, the pull-back maneuver-assisted insertion led to significantly higher ECAP thresholds in the basal cochlear electrode channels (Electrode 2–Electrode 3) than with conventional insertion. Data are means ± standard error of mean (SEMs), *P < 0.05 by independent t-test.

After final intracochlear positioning of the electrode arrays, electrically evoked compound action potential (ECAP) thresholds were measured in every channel for all subjects using automatic neural response telemetry (NRT) intraoperatively. Intraoperative ECAP thresholds varied across electrode arrays, regardless of the insertion technique (Fig. 3). We observed that the ECAP thresholds tended to gradually increase from the apical to basal cochlear region, with disproportionately high threshold spikes from the last three basal electrodes (E1-3). The pull-back maneuver led to significantly lower ECAP thresholds, only in the upper basal and middle cochlear electrode channels (E6-E12), compared with the conventional group (*P* < 0.05 for E6-E12 by independent t-test) (Fig. 3). These electrophysiological findings highlight the effect of the pull-back maneuver on enhancing modiolar proximity, which was greatest for the upper basal and middle turns of the cochlea. Meanwhile, the pull-back technique inevitably led to significantly higher ECAP thresholds in the lower basal cochlear electrode channels (E2-3) than those with conventional insertion (E2, *P* = 0.005 and E3, *P* < 0.001 by independent t-test, respectively). Overall, significant decrease in electrophysiological thresholds, especially in the upper basal and middle electrodes, indicate that the electrode array is placed closer to the modiolus when the pull-back maneuver is employed, which is congruent with the significantly reduced spiral diameter of slim modiolar electrodes following the pull-back maneuver.

**Figure 4:**
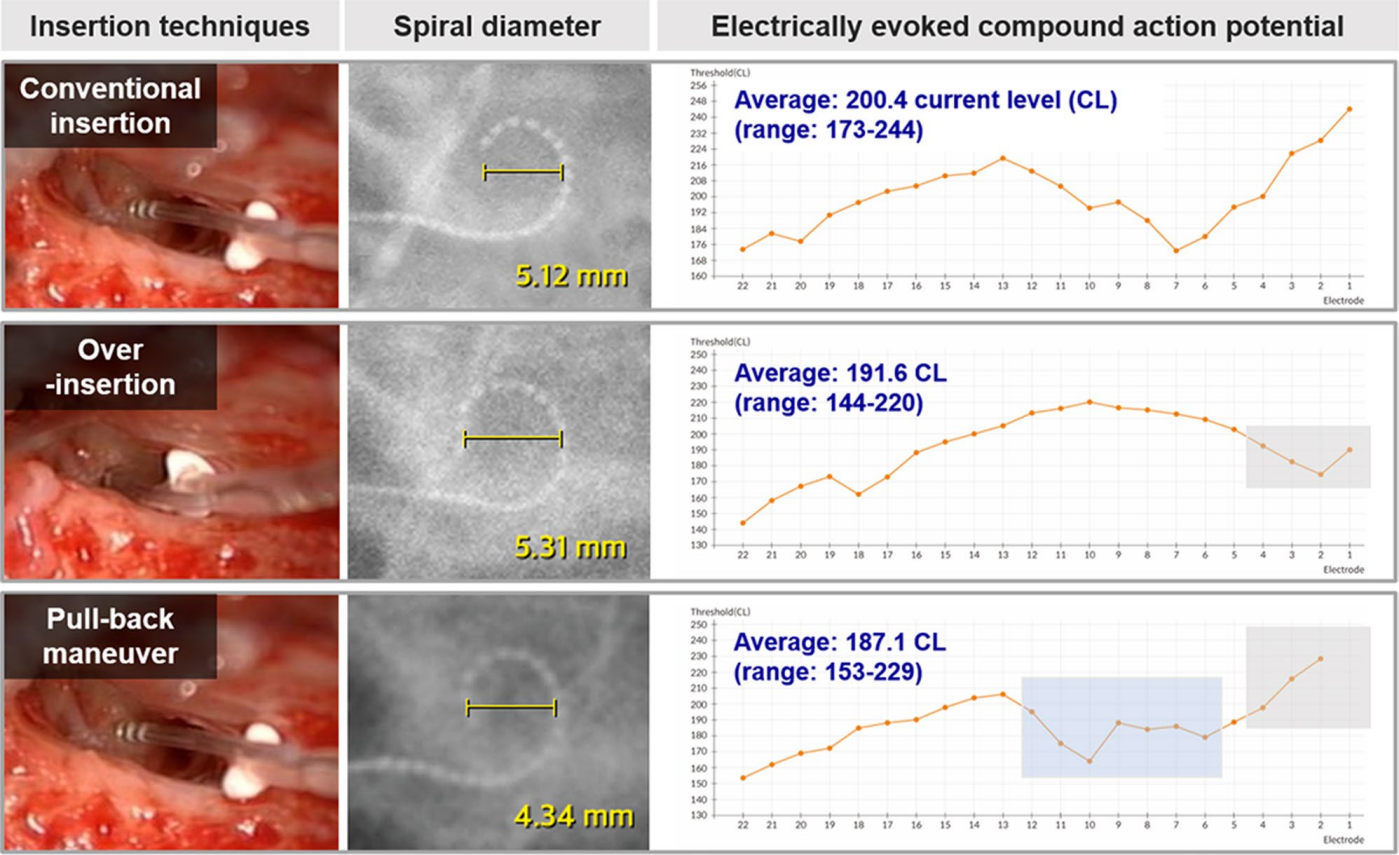
Serial changes in the spiral diameter of the slim modiolar electrode array, and electrically evoked compound action potential threshold during three insertion techniques. A representative case showing a differential spiral diameter depending on the insertion technique. This female patient is 23 months old at the time of examination and manifested bilateral deafness with congenital onset. The slim modiolar electrode arrays can be advanced or retreated with reference to three white markers of the electrode array and their relationship with the round window, facilitating a finely tuned insertion technique. Specifically, the pull-back maneuver-assisted insertion led to a significantly shorter spiral diameter based on intraoperative simple X-ray (i.e., cochlear view), compared with conventional and over-insertion. However, the angular insertion depth remained unchanged even after the pull-back maneuver-assisted insertion, compared with over-insertion. During the procedure, over-insertion led to reduced ECAP thresholds of the last four basal electrodes (Electrode 1–Electrode 4, gray rectangular box), compared with conventional insertion. Subsequently, pulling-back the electrode significantly reduced thresholds for the upper basal and middle electrodes (Electrode 6–Electrode 12, blue rectangular box), while again elevating ECAP thresholds of the last four basal electrodes (Electrode 1–Electrode 4, gray rectangular box) similar to the those of conventional insertion.

Supported by the ECAP threshold configurations obtained in the current study, and also by the previous report on the effect of the pull-back maneuver on modiolar proximity of slim modiolar electrodes in human cadaveric temporal bones^25^, we then measured ECAP thresholds and took simple X-ray to check spiral diameters in all three techniques of insertion in a single subject: conventional insertion of the electrode first, then over-insertion, followed by pulling back (Fig. 4). Over-insertion led to reduced ECAP thresholds of the last four basal electrodes, taking elevated thresholds for upper basal and middle electrodes as a trade-off. Notably, pulling-back the electrode significantly reduced thresholds for the upper basal and middle electrodes, while again elevating ECAP thresholds of the last four basal electrodes. The decreased thresholds of the upper basal and middle electrodes were in accordance with the smallest spiral diameter related to the pull-back maneuver in this subject**,** replicating the effect of the pull-back maneuver-assisted insertion.

### Intraoperative and postoperative impedances

**Figure 5:**
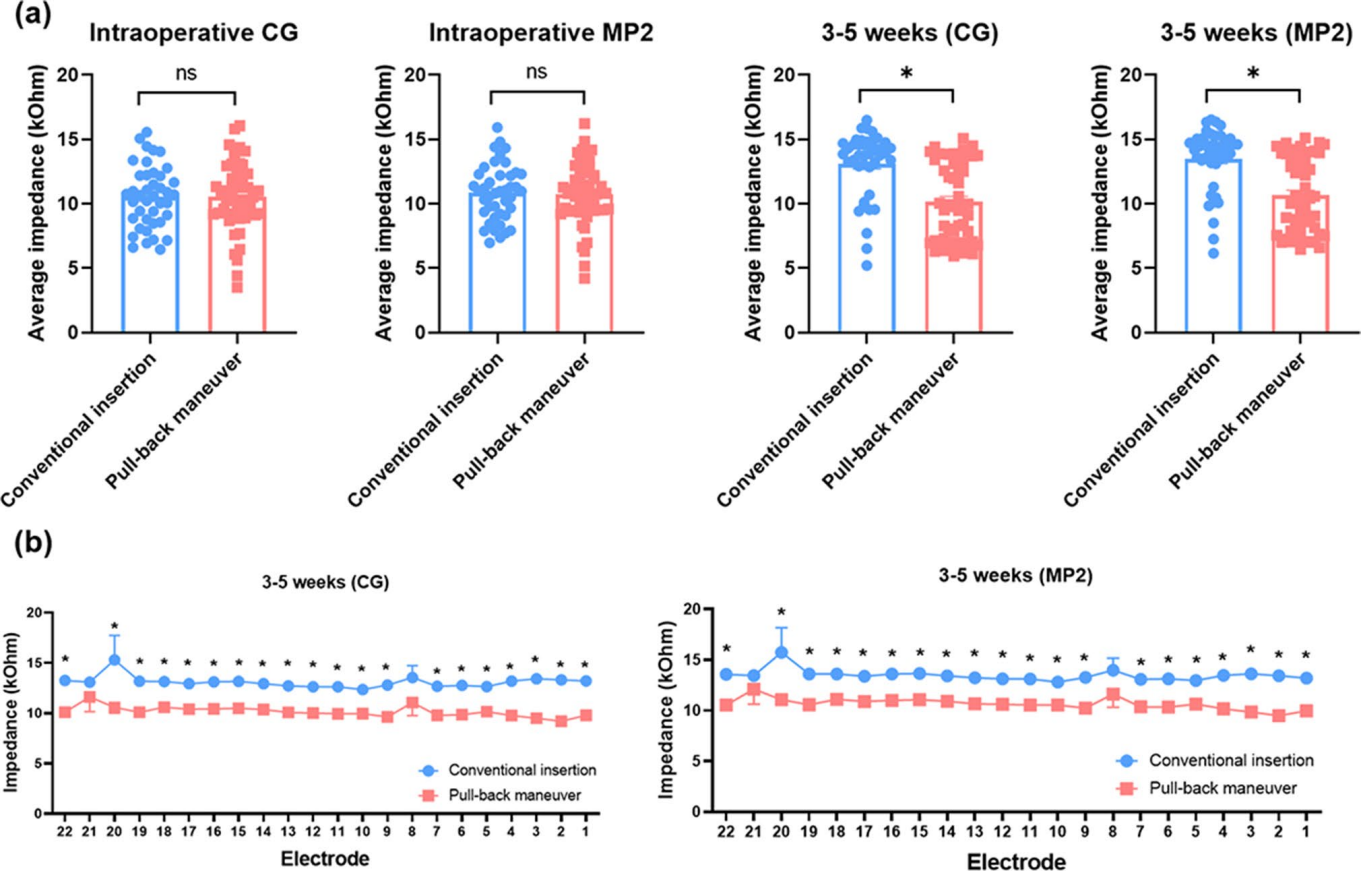
Comparison of intraoperative and postoperative impedance across electrode contacts between the conventional insertion and pull-back maneuver groups for cochlear implant recipients with slim modiolar electrode. (**a**) Average impedance values using monopolar (MP2) and common ground (CG) stimulation configurations intraoperatively and postoperatively. (**b**) Postoperative impedance per electrode contact using MP2 and CG modes. Data are means ± standard error of mean (SEMs). *Statistical significance.

We further analyzed the impedance of electrode contacts according to insertion techniques: conventional insertion and pull-back maneuver. The comparison of average and individual electrode contact impedances, depending on insertion techniques, are shown in Fig. 5. Regardless of stimulation strategies using monopolar (MP2) and common-ground (CG) configurations, no difference in impedances was observed between conventional insertion and pull-back maneuver intraoperatively. By contrast, the electrode repositioning by the pull-back maneuver, a closer modiolar placement of the slim modiolar electrode led to significantly lower impedances across electrodes than conventional insertion postoperatively (approximately 3–5 weeks).

### Speech perception outcomes

**Figure 6:**
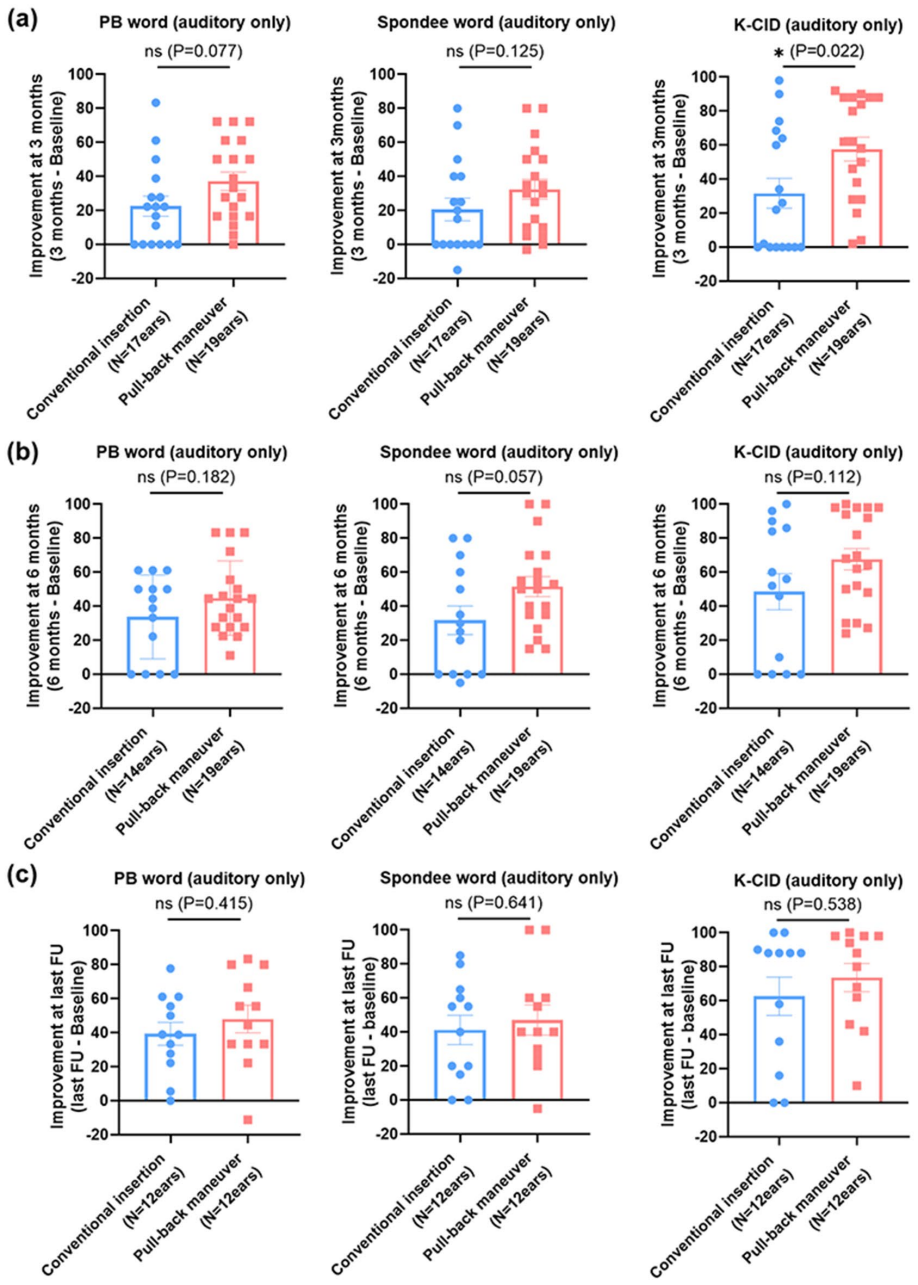
Comparison of serial changes in speech evaluation scores between the conventional insertion and pull-back maneuver groups in adult cochlear implant recipients (> 15-years old). The improvement refers to the difference in speech perception scores between preoperative (baseline) and postoperative three timepoint. (**a**) The improvement of word scores was seemingly higher in the pull-back maneuver group than in the conventional insertion group, but the difference did not reach a level of significance. However, a significant improvement of sentence scores (i.e., K-CID) during the postoperative 3-month follow-up was found in the pull-back maneuver group compared to that in the conventional insertion group. (**b**, **c**) The pull-back maneuver tended to elicit better speech perception in adult cochlear implantees compared to conventional insertion at 6 months and last follow-up postoperatively, although the effect was not statistically significant. The last follow-up refers to the speech evaluation test conducted between 6 and 12 months.

We also explored if the pull-back maneuver-based physical advantage can lead to actual improvement in speech outcome of adult cochlear implant recipients. To do so, adult cochlear implant recipients (> 15 years) were subclassified into two groups based on the insertion techniques (conventional insertion versus pull-back groups) and speech perception improvements between baseline and 3 and 6 months, and last follow-up (between 6 months and 1 year) postoperatively were compared. Preoperative speech evaluation was based on the test score with hearing aids considering the implanted ear only (Fig. S1). Considering the obviously poor but still significantly better preoperative Spondee word and Korean central institute for deafness (K-CID) scores in the pull-back group than in the conventional group (*P* = 0.022 and *P* = 0.003 by Mann–Whitney U test, respectively) (Fig. S1), we decided to compare the speech perception improvements between baseline and throughout follow-up periods up to 12 months (Fig. 6) as well as making a direct comparison of absolute values of speech perception score (Fig. S1) to minimize the confounding effect resulting from preoperative hearing aid performance. As expected, the pull-back group showed a significantly tighter spiral configuration (i.e., reduced spiral diameter) than the conventional insertion group (Fig. S2). However, age at cochlear implant, sex, cochlear duct length, and AID did not significantly differ between the two groups (Fig. S2).

Baseline and three-month postoperative speech perception evaluation were available for 36 ears (conventional insertion, 17 ears; pull-back maneuver, 19 ears) (Fig. 6a). Importantly, the pull-back maneuver elicits a significantly greater improvement of speech evaluation in the K-CID score at postoperative 3 months compared with that in the conventional insertion group (*P* = 0.022 by Mann–Whitney U test) (Fig. 6a). Furthermore, the pull-back maneuver tended to yield a greater improvement in the 3-month postoperative word scores (phonetically balanced [PB] word, *P* = 0.077 by Mann–Whitney U test; Spondee word, *P* = 0.125 by Mann–Whitney U test), which was not statistically significant. These results may exclude the hypothesis that speech outcome improvement due to reduction of EMD may have a saturation at the level of EMD of the conventional perimodiolar electrode. However, the in vivo effects of the pull-back maneuver on speech outcomes would confer more clinical significance in the early stage after surgery. For example, at 6 months postoperatively, the statistical significance for greater speech improvement in the pull-back group was not observed, even though the pull-back maneuver still showed a tendency of greater improvement of spondee word scores compared with the conventional insertion (Fig. 6b). Finally, we noted no difference in speech perception scores in all domains at the most recent follow-up sometime between 6 months and 1 year (median, 10 months; range 8–12 months) (Fig. 6c), suggesting that superiority of the pull-back maneuver on speech outcomes over the conventional insertion group is likely to be diluted over time. It could be that the greater improvement in speech outcome due to better modiolar proximity may reach saturation after a certain amount of follow-up time, and after that time point, there would be no significant difference in speech improvement between two insertion methods.

In a bid to cross-check and strengthen our argument that this pull-back maneuver yields greater speech improvement at postoperative 3 months, we further investigated whether the spiral diameter that reflects modiolar proximity correlates with improvement in the speech outcome of adult cochlear implant recipients (> 15 years). In parallel with observations from Fig. 6, spiral diameter inversely correlates with open-set sentence recognition (K-CID), in the early postoperative stage (Fig. S3). However, this correlation lost its statistical significance in all domains as the follow-up period became longer.

### Battery consumption

The parameters reflecting battery consumption, including standard, standard rechargeable, compact rechargeable, did not significantly differ among the groups in all domains (standard, *P* = 0.67; standard rechargeable, *P* = 0.94; compact rechargeable, *P* = 0.56) (Fig. S4).

## Discussion

To the best of our knowledge, this is the first study to demonstrate the in vivo effects of the pull-back maneuver, coupled with slim modiolar electrodes, on speech outcomes, EMD and electrophysiological parameters. Adjusting the marker position in and out across the round window using this surgical maneuver affected the electrode position mediolaterally without advancing the tip of it anteriorly, replicating those of previous ex vivo studies^4,25^. As illustrated in Fig. 2b, the pull-back maneuver led to better modiolar proximity as demonstrated by a significantly reduced spiral diameter on X-ray while maintaining the depth of angular insertion. Further, several studies have elaborated a significant correlation between EMD and electrical stimulation levels, implying electrophysiological effects of placing electrode array latero-medially^32–36^. Similarly, a significant decrease in electrophysiological thresholds, coupled with reduced spiral diameters, clearly indicates that the electrode array is placed closer to the modiolus if the pull-back maneuver is employed.

Which cochlear regions would benefit from electrode repositioning by the pull-back maneuver? The observed effect of the electrode position mediolaterally on the neuronal spread of excitation has extensively been reported for conventional perimodiolar electrodes^27,30,37^. Specifically, Basta et al. suggested that the pull-back technique has its greatest effect exclusively in the basal region of the cochlea (i.e., E5) for conventional perimodiolar electrodes (e.g., Nucleus 24 Contour Advanced electrode array), as evidenced by the significantly lower spread of excitation and increased frequency discrimination in the basal electrode (i.e., E5) than those in the middle electrodes (i.e., E10 and E15)^27^. To interest, our current study elucidates that pull-back maneuver-assisted insertion of slim modiolar electrodes exerts a slightly different effect on electrophysiological parameters than those of conventional perimodiolar electrodes. Specifically, as presented herein, ECAP thresholds of electrode channels corresponding to the upper basal and middle turns (E6-E12) most significantly dropped down when aided by the pull-back maneuver compared with the conventional insertion. This electrophysiologic data was supported by our recent radiological observation that modified ICPI, a reflection of the distance of each electrode contact relative to the modiolus at specific points, marked the lowest value at the upper basal and middle turn regions of cochlear implantees with pull-back maneuver-assisted insertion of slim modiolar electrodes^31^. Different tonotopic effect from the same maneuver between conventional perimodiolar and slim modiolar electrodes would be attributed to differences in electrode array size, inter-electrode contact distance, and electrode properties (e.g., elasticity). Although the ECAP thresholds of the most basal cochlear electrode channels (E2-3) were relatively higher with pull-back maneuver–assisted insertion than with conventional insertion, as if it were a trade-off, the clinical significance of the loss of modiolar proximity in the distal basal region (E2-3) still requires further research.

Previously, Holden et al. suggested an indicator evaluating the medial–lateral position of electrode array (i.e., wrapping factor) and confirmed that a closer modiolar placement of electrode array remained positively correlated with outcome in postlingually deaf adults, even after adjustment of cofactors^13^. In addition, a generalized linear model has recently shown that the average EMD was one of the most significant factors to predict speech perception scores in cochlear implant recipients with pre-curved arrays^12^. Our study merits special attention in a sense that further improvement of modiolar proximity by the pull-back maneuver, even at the enhanced level of EMD provided by slim modiolar electrodes compared with conventional perimodiolar electrodes, would again lead to better speech perception outcomes, especially at least in the early stage after surgery, in adult cochlear implant recipients.

It could be that improved electrophysiological parameters coupled with enhanced modiolar proximity accounts for the observed functional benefit related to the pull-back maneuver in our study. Indeed, a higher mean ECAP threshold was previously found to be associated with worse speech recognition scores for cochlear implant recipients with slim modiolar electrode^23^. Additionally, Holder et al. reported that lateral wall electrodes showed higher impedances than the slim modiolar electrode postoperatively^1^, probably due to greater distance between the electrode and neural tissues^38^. Consistent with these findings, impedance levels in the pull-back maneuver group presented herein were significantly lower postoperatively compared with those from the conventional insertion group among implantees with slim modiolar electrodes.

Theoretically, impedance levels confer messages also on the status of the electrode-fibrous tissue formation or changes to the resistive properties of the surrounding fluid and tissue^1^. Notably, the recipients with lateral wall electrode showed not only the higher absolute value but also more variation in impedances over time. Specifically, Shaul et al. showed that lateral wall electrodes significantly led to increase in impedance spikes, related to immunological reaction in the inner ear, compared with the pre-curved arrays^39^. These results suggest that the physical location of electrodes could affect impedances in several ways. Of course, higher impedance has been proposed to reduce the dynamic range of the stimulation, while requiring higher voltage across the electrode-tissue interface. Taken together, the impedance reduction provided by the pull-back maneuver could be a result of less vulnerability/high tolerance to tissue reaction and would in turn help to formulate mapping strategy, thus enhancing the functional benefits as evidenced by early stages of the sentence domain.

Intriguingly, our data showed significantly higher speech perception scores at 3 months postoperatively, albeit only in the sentence domain, for the pull-back maneuver group. Particularly, if context clues were provided, the focused electrical stimulation by the pull-back maneuver together with the top-down processing may have improved the sentence score. Unfortunately, this observation was not replicated in the words score domain. Furthermore, improved EMD by the pull-back maneuver on speech perception outcomes has failed to demonstrate long-term functional improvement. This finding is not different from those in previous studies^35,40^, perhaps due to the presence of confounding factors and small sample sizes limiting the power to identify statistically significant differences. Alternatively, audiological rehabilitation and central brain plasticity may also have diluted the advantage from the improved EMD, resulting in the similar functional outcomes over time between groups, as already shown in several other studies^41^. Given this, focusing on short-term improvements in speech perception might be a better strategy for deciphering the in vivo effect of the pull-back maneuver-assisted insertion of slim modiolar electrodes on speech perception. In a situation where the importance of EMD can be more pronounced such as cochlear nerve hypoplasia or reduced number of spiral ganglion cells, the effect of this pull-back maneuver might be sustained for a longer time.

Although MR imaing indicates cochlear nerve agenesis, such cases may have extremely low, but still exist residual spiral ganglion neurons (SGNs) near the modiolus^42^. Based on the evidence that the electrode-modiolus distance is closely correlated with the ECAP thresholds of the electrodes including slim modiolar electrodes^43^, a better modiolar proximity with the pull-back maneuver would potentially lead to more favorable outcomes in those with cochlear nerve deficiency than those requiring conventional insertion. In other words, slim modiolar electrodes with pull-back maneuvers could be better in cases with cochlear nerve deficiency, if not completely defective modiolus; however, future studies are required to fully elucidate the effects of modiolar proximity on outcomes among cochlear implant recipients with cochlear nerve deficiency.

There are limitations in this study that should be addressed in the future. Our current study was designed to be a cross-sectional evaluation, which, along with the retrospective study design, may weaken the clinical implications of the results. In addition, selection bias and pre-conceived bias of what was happening would limit the generalization of our results. Future studies employing randomization and correction for multiple comparisons in large-scale cases would be the best fit for proving effects of the pull-back maneuver on electrophysiological and audiological outcomes after cochlear implantation. For the first time, we showed the clinical effect of the pull-back technique using slim modiolar electrodes on speech perception scores, thus strengthening the importance of modiolar proximity of slim modiolar electrodes. However, the results were limited by the short-term follow-up and relatively small number of study subjects consisting of only recipients with post-lingual deafness. Finally, confounding variables related to the final position of the electrode arrays were minimized, but not eliminated. The subjects enrolled in this study had relatively heterogeneous etiologies of deafness. Thus, more efforts to minimize confounding factors are necessary to draw firm conclusions. Future studies with more strict matching strategies should also be considered.

## Conclusion

We, for the first time, demonstrated the advantages and safety of the pull-back maneuver-assisted insertion of slim modiolar electrodes in adult cochlear implantees, which include maximizing modiolar proximity, better electrophysiological findings, and improved open-set sentence recognition at least in the early stage after implant surgery compared with the conventional insertion.

## Materials and methods

### Participants

This retrospective study was conducted from July 2018 to December 2019 at Seoul National University Bundang Hospital. Subjects with the following conditions were excluded from this study: (1) definite retro-cochlear pathology (e.g., cochlear nerve agenesis) on radiological imaging, (2) history of explantation due to device failure, and (3) severe craniofacial syndrome. This study was approved by the Seoul National University Bundang Hospital Institutional Review Board (IRB No. B-2020-869) and was conducted in accordance with the principles of the Declaration of Helsinki. We obtained written informed consent from either the parents of children or participants themselves in this study.

### Insertion techniques

Using a sheath-based deployment system^4^, the electrode arrays can be advanced or retreated in reference to three white markers of the electrode array and their relationship with the round window, facilitating a finely tuned insertion technique: conventional insertion (first white marker) (Fig. 1a), over insertion (up to third white marker) (Fig. 1b), and pull-back maneuver assisted insertion (first white marker) (Fig. 1c). The insertion technique was decided entirely in chronological order. The conventional insertion refers to the initial insertion of the electrode array up to the first white marker as outlined by the product manual (Cochlear; Sydney, Australia). As for over-insertion, the electrode was inserted a little bit more deeply up to the third white marker, advancing past the first marker. As described by Riemann et al.^25^, the pull-back maneuver assisted insertion relies on pulling back until the first white marker of the electrode becomes visible again in the round window, following over-insertion.

### Spiral diameter and angular depth of insertion

The depth of angular insertion made by the slim modiolar electrodes in situ on transorbital X-ray were compared between different insertion techniques by an otorhinolaryngologist (Huh, J) who was blind to the insertion technique. As previously described^31^, upon transorbital X-ray which is taken on the day after implantation, the spiral diameter of the electrode turn was defined as the distance of the electrode turn measured on a horizontal line across the modiolus. As for a single subject in Fig. 4, X-ray with 45° side oblique view is taken for checking the status of inserted electrode intraoperatively. However, this intraoperative oblique view potentially could result in errors in measuring electrode-to-modiolus distance due to inconsistency in the acquisition angle of the X-ray.

### Measurement of electrically evoked compound action potential thresholds and impedances

After final intracochlear positioning of the electrode arrays, telemetry recordings were made under sterile conditions in the operating field. As a reference to telemetric measurement via NRT, ECAP thresholds were measured in every channel for all subjects. Only when we were able to obtain ECAP responses from at least nineteen electrodes, the NRT thresholds of such subjects were used for comparison. The threshold of electrodes which did not elicit any NRT response even at the maximum stimulus was judged as 255 (CL) for statistical analyses. Further, stimulation rate (Hz) and the number of sweeps were set as 250 Hz, and 35, respectively, as outlined by guideline.

Using the Cochlear custom sound EP 5.2 software, impedances were determined with MP2 and CG stimulation configurations using biphasic pulses at a rate of 250 Hz and were measured intraoperatively and postoperatively (approximately 3–5 weeks). During the test, Custom Sound EP software stimulates each electrode at 80 Current Level in postoperative and intraoperative testing, using a pulse width of 25 us.

### Cochlear duct length measurement

As described in our previous study^43^, a readily available surgical pre-planning tool (OTOPLAN Version 2.0 by MED-EL Innsbruck, Austria, www.otoplan.ch) used the Elliptic-Circular Approximation (ECA) method^44^ was employed to determine individual cochlear duct length from all our subjects.

### Audiological evaluation

As previously described^45,46^, speech performance was thoroughly assessed preoperatively, and 3, 6, and 10–12 months after cochlear implantation using Korean version of the Central Institute for the Deaf (K-CID) and the Korean Spondees and phonetically balanced (PB) words by experienced speech-therapists. The Korean Central Institute for Deafness (K-CID) test, designed to assess understanding of speech performance in everyday conversational situations, was scored based on the percentage of correctly identified words. Speech perception performance was also evaluated through word-recognition tasks using Spondees and phonetically balanced (PB) words at 70 dB SPL in sound-field under a condition of audio-only without any visual cues. Subjects were instructed to repeat the speech stimulus verbally, and the score was recorded as the percent correct (%). The speech perception performance was based on the test score only for each implanted ear while the contralateral ear was masked or plugged when necessary. Specifically, plugging or masking for better ear was performed in cases with asymmetric sensorineural hearing loss between two ears. In case of severe-to-profound sensorineural hearing loss in both ears, plugging or masking was selectively performed in better ears if they had meaningful residual hearing that affects the performance in the implanted ears.

Indeed, there are differences in the test battery of speech evaluation, depending on the age of 15 years (cut-off value) here in South Korea. Specifically, the perception tests, including PB word, Spondee word, and K-CID Sentence, were conducted in those over 15 years, while only PB word and Sentence tests were conducted in participants below 15. In addition, most cochlear implant recipients with post-lingual onset of deafness were older than 15 years in this cohort. Thus, we explored whether the pull-back maneuver-based physical advantage can lead to improvement in speech outcomes of adult cochlear implant recipients (> 15 years).

### Statistical analyses

The data are represented as the mean ± SEM. All analyses were done and illustrated using the R statistical package (version 3.3.2, R Foundation for Statistical Computing, Vienna, Austria). Again, all the analyses employed and illustrated used the GraphPad Prism version 9.0.0 for Windows, GraphPad Software, San Diego, California USA (www.graphpad.com). To compare the outcomes depending on the insertion technique, an independent t-test was performed if the values were normally distributed. Alternatively, the Mann–Whitney U test to obtain comparable results, as appropriate, if the variables were not normally distributed. *P* values < 0.05 were considered statistically significant.

## Supplementary Information

Supplementary Figure S1.

Supplementary Figure S2.

Supplementary Figure S3.

Supplementary Figure S4.

## Data Availability

Data for all submitted results is available.
