# Supplementary material for: Effects of in vivo repositioning of slim modiolar electrodes on electrical thresholds and speech perception
Source: Sci Rep. 2021 Jul 23;11:15135. doi: 10.1038/s41598-021-94668-6 (PMC8302625; doi:10.1038/s41598-021-94668-6)
Supplement: Supplementary file 1 — Supplementary Figure S1. [file 41598_2021_94668_MOESM1_ESM.pdf]

Effects of *in vivo* repositioning of slim modiolar electrodes on electrical thresholds and speech perception

Sang-Yeon Lee, Young Seok Kim, Hyung Dong Jo, Yoonjoong Kim, Marge Carandang, Gene Huh, Byung Yoon Choi

Fig.S1

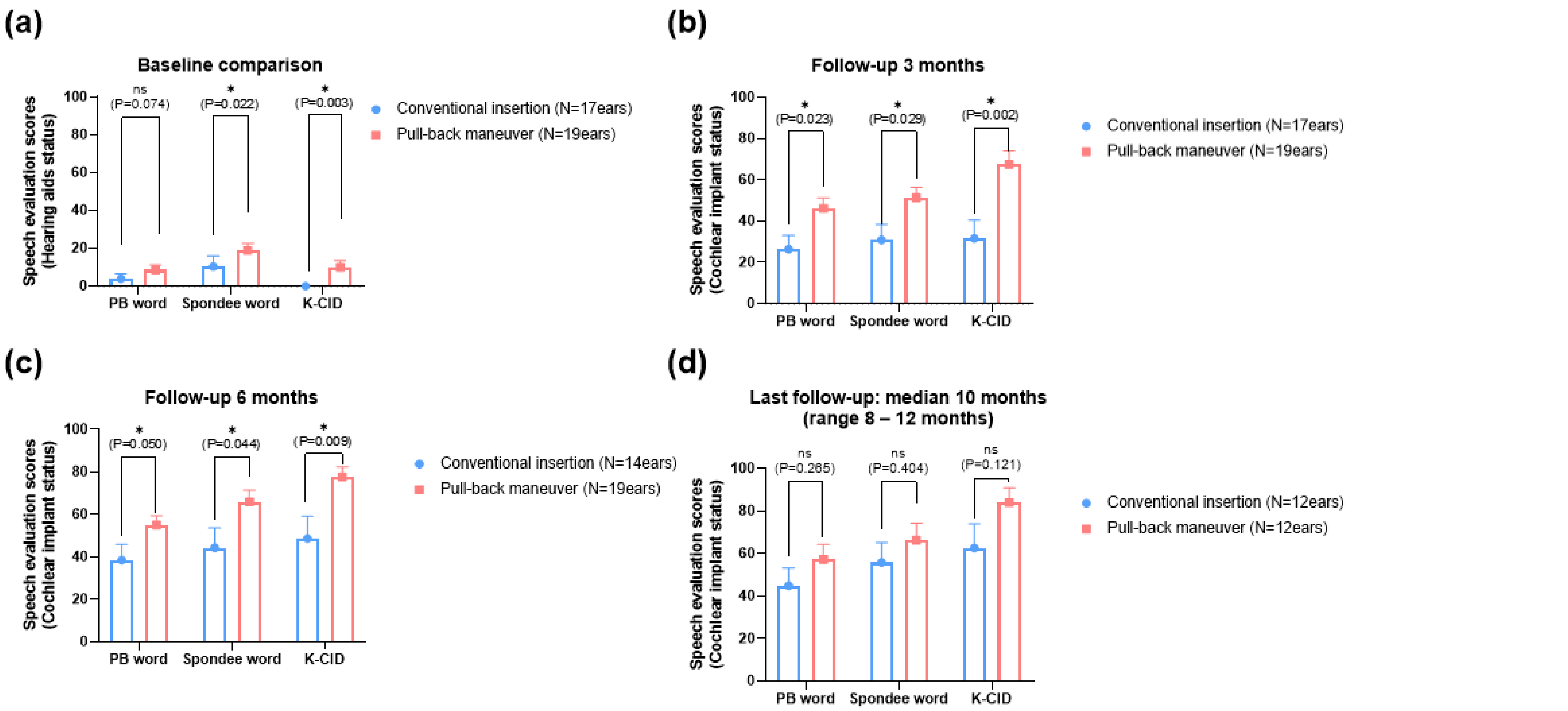

Supplemental figure 1. (a) Preoperative speech perception scores between conventional and pull-back groups. The preoperative speech perception scores are based on the testing result when wearing a hearing aid in the implanted ear. No difference in preoperative phonetically balanced (PB) word scores was observed between two groups. However, pull-back maneuver group had a significantly higher Spondee word and Korean central institute for deafness (K-CID) scores as well as PB word scores, than did the conventional insertion group. (b-d) Postoperative speech perception scores at each follow-up period between conventional and pull-back groups. Data are means ± standard error of mean (SEMs). * $P<0.05$ by independent t-test or Mann-Whitney U test depending on the normal distribution of data. ns, no statistical significance.
