# Supplementary material for: Effects of in vivo repositioning of slim modiolar electrodes on electrical thresholds and speech perception
Source: Sci Rep. 2021 Jul 23;11:15135. doi: 10.1038/s41598-021-94668-6 (PMC8302625; doi:10.1038/s41598-021-94668-6)
Supplement: Supplementary file 2 — Supplementary Figure S2. [file 41598_2021_94668_MOESM2_ESM.pdf]

Effects of *in vivo* repositioning of slim modiolar electrodes on electrical thresholds and speech perception

Sang-Yeon Lee, Young Seok Kim, Hyung Dong Jo, Yoonjoong Kim, Marge Carandang, Gene Huh, Byung Yoon Choi

Fig.S2

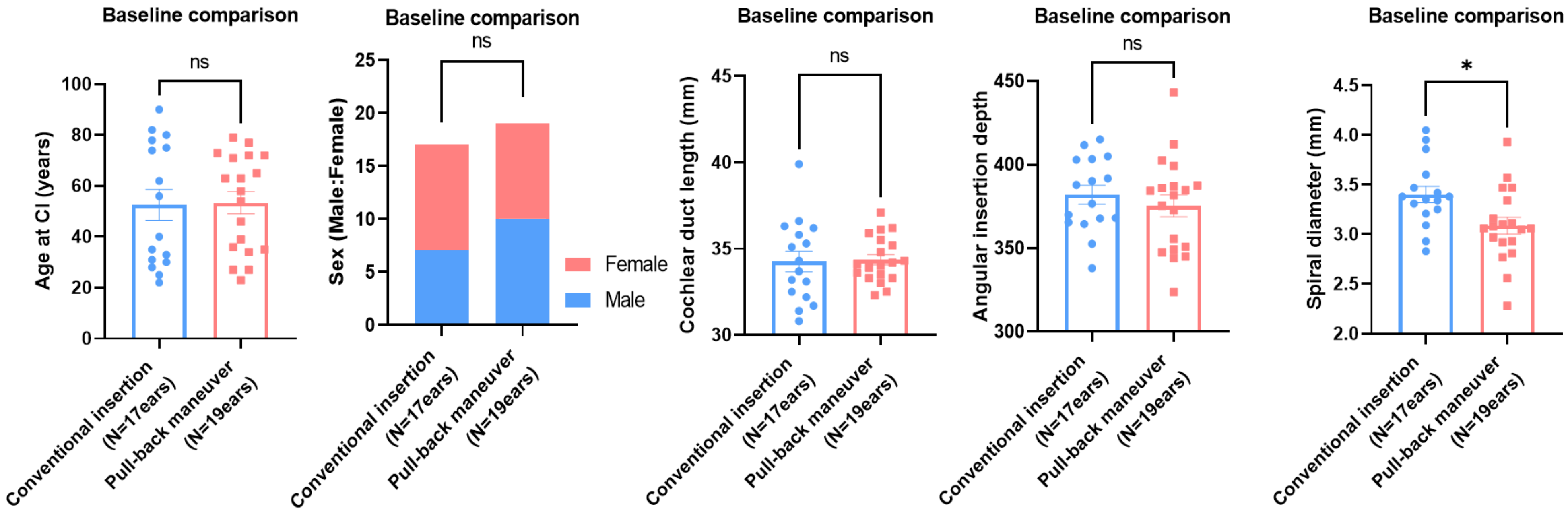

Supplemental figure 2. Demographic and clinical data in this figure included only adult cochlear implant recipients (> 15 years). According to the baseline evaluation, there were no significant differences in age at cochlear implantation and sex between the conventional insertion and pull-back groups. Further, neither angular insertion depth (AID) nor cochlear duct length (CDL) was different between two groups. Expectedly, the pull-back maneuver group had a significantly shorter spiral diameter, reflecting better modiolar proximity, than did the conventional insertion group. Data are means ± standard error of mean (SEMs). * $P<0.05$ by independent t-test. ns, no statistical significance.
