# Supplementary material for: Effects of in vivo repositioning of slim modiolar electrodes on electrical thresholds and speech perception
Source: Sci Rep. 2021 Jul 23;11:15135. doi: 10.1038/s41598-021-94668-6 (PMC8302625; doi:10.1038/s41598-021-94668-6)
Supplement: Supplementary file 3 — Supplementary Figure S3. [file 41598_2021_94668_MOESM3_ESM.pdf]

Effects of *in vivo* repositioning of slim modiolar electrodes on electrical thresholds and speech perception

Sang-Yeon Lee, Young Seok Kim, Hyung Dong Jo, Yoonjoong Kim, Marge Carandang, Gene Huh, Byung Yoon Choi

Fig.S3

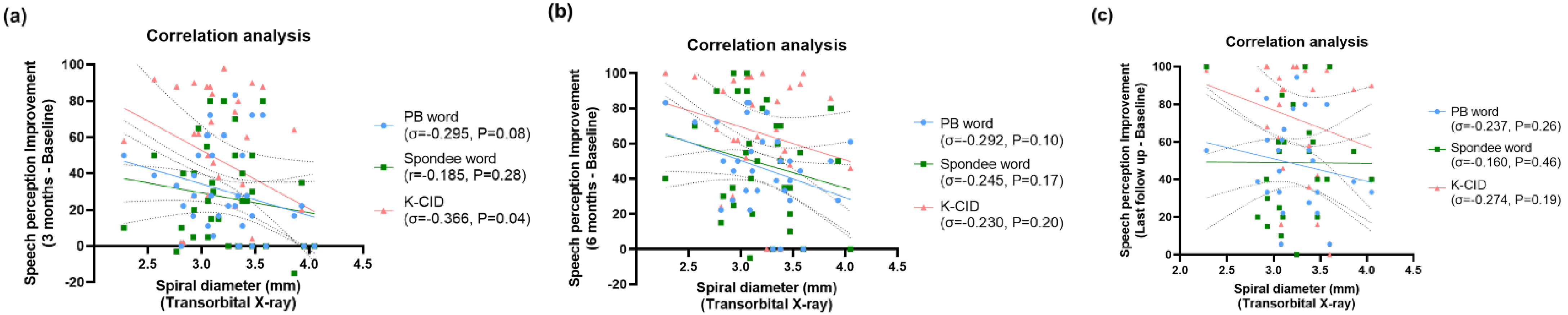

Supplemental figure 3. Correlation analyses between speech perception outcomes and spiral diameter based on transorbital X-ray. (a) The spiral diameter significantly correlates with improvement between the baseline and 3 months speech evaluation, only in the Korean central institute for deafness (K-CID) score. (b-c) there were no significant correlations between the spiral diameter and speech perception improvement thereafter on PB word and phonetically balanced word. All statistics, except for the correlation between the improvement of the spondee word during the first three months and the spiral diameter, were performed using Spearman’s rank-order correlation analyses.
