# Supplementary material for: Effects of in vivo repositioning of slim modiolar electrodes on electrical thresholds and speech perception
Source: Sci Rep. 2021 Jul 23;11:15135. doi: 10.1038/s41598-021-94668-6 (PMC8302625; doi:10.1038/s41598-021-94668-6)
Supplement: Supplementary file 4 — Supplementary Figure S4. [file 41598_2021_94668_MOESM4_ESM.pdf]

Effects of *in vivo* repositioning of slim modiolar electrodes on electrical thresholds and speech perception

Sang-Yeon Lee, Young Seok Kim, Hyung Dong Jo, Yoonjoong Kim, Marge Carandang, Gene Huh, Byung Yoon Choi

Fig.S4

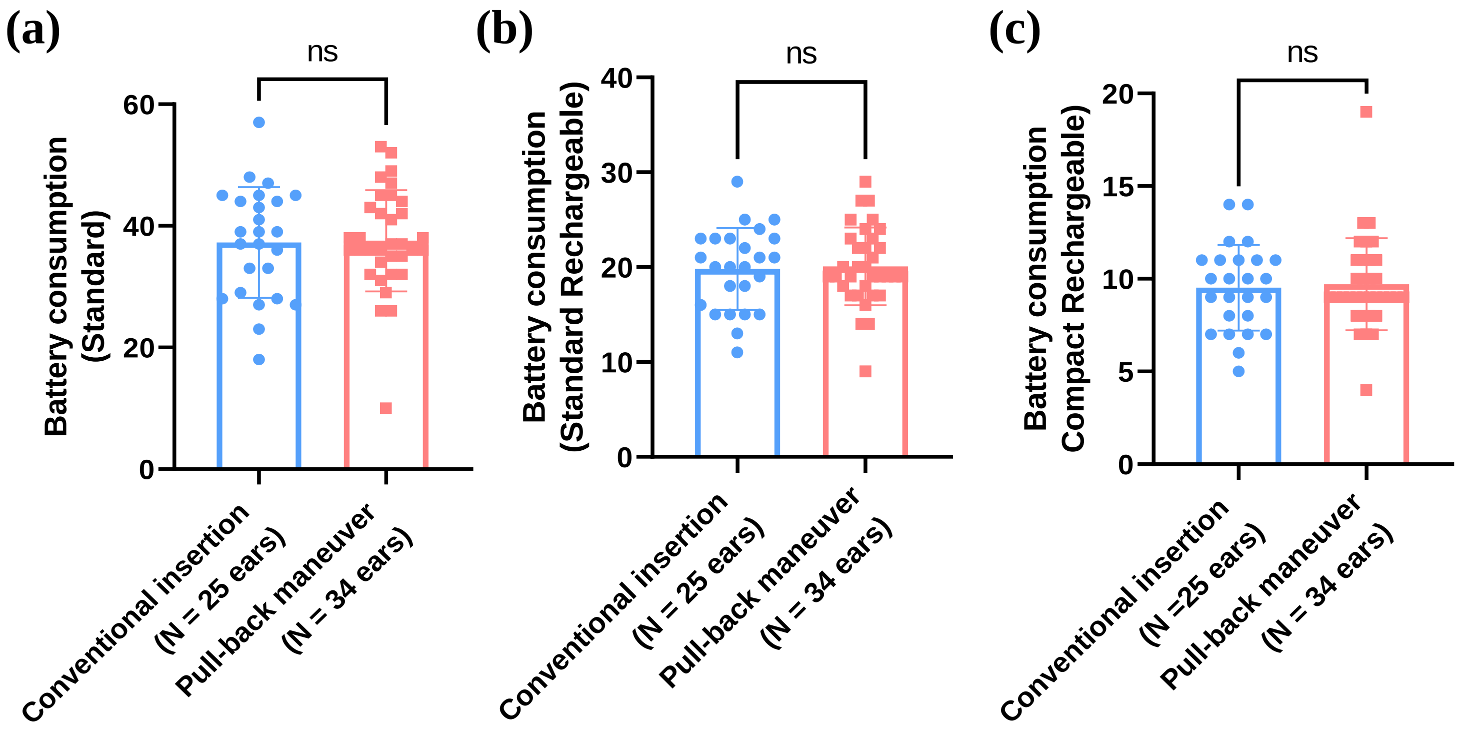

Supplemental figure 4. Comparison of battery consumption between groups. No statistical significance on battery consumption, in terms of (a) Standard, (b) standard rechargeable, (c) compact rechargeable, was observed, regardless of the insertion technique. ns, no statistical significance.
